# A Minimal Model for Multiple Epidemics and Immunity Spreading

**DOI:** 10.1371/journal.pone.0013326

**Published:** 2010-10-18

**Authors:** Kim Sneppen, Ala Trusina, Mogens H. Jensen, Stefan Bornholdt

**Affiliations:** 1 Niels Bohr Institute/CMOL, Copenhagen, Denmark; 2 Institute for Theoretical Physics, University of Bremen, Bremen, Germany; University of Leeds, United Kingdom

## Abstract

Pathogens and parasites are ubiquitous in the living world, being limited only by availability of suitable hosts. The ability to transmit a particular disease depends on competing infections as well as on the status of host immunity. Multiple diseases compete for the same resource and their fate is coupled to each other. Such couplings have many facets, for example cross-immunization between related influenza strains, mutual inhibition by killing the host, or possible even a mutual catalytic effect if host immunity is impaired. We here introduce a minimal model for an unlimited number of unrelated pathogens whose interaction is simplified to simple mutual exclusion. The model incorporates an ongoing development of host immunity to past diseases, while leaving the system open for emergence of new diseases. The model exhibits a rich dynamical behavior with interacting infection waves, leaving broad trails of immunization in the host population. This obtained immunization pattern depends only on the system size and on the mutation rate that initiates new diseases.

## Introduction

Spreading of infectious diseases occurs at all levels of life, ranging from viruses preying on bacteria [1]–[6] to a diversity of pathogens preying on plants [7], [8], animals [9], or humans [10]–[14]. Immunization is perhaps the single most important process that allows complex life to survive this near-infinity of pathogens in our world. Mutations constantly provide a supply of new pathogens that can bypass previously developed defense mechanism of their hosts, and as a result there is an ongoing flux of new diseases that attempt to propagate on any host species. Seen from a complex systems point of view, the ongoing battle between pathogens and the immune systems of their hosts suggests a new class of dynamics, where “new” replaces “old” irreversibly.

When modeling the propagation of diseases it is not important whether the host becomes sick. What matters is the likelihood of spreading to new hosts before the current host either dies or develops immunity. Going beyond death and other interference mechanisms between diseases [15], [16], a more direct interaction between diseases are observed between influenza epidemics where mutual inhibition is obtained by cross-immunity [17]–[21]. Such cross-immunizations are in particular important between closely related diseases, and a main objective in the associated modeling of influenza spreading is to understand the relatively small sustained diversity there is between various strains of related influenzas.

The present paper does not aim to include any effect of cross-immunization, an approximation that implicitly ignores/coarse grain over disease differences on the level of strain variations. Also we ignore possible catalytic effects between diseases, effects that would be expected when diseases weaken the immune system. Further we simplify the ecological interference by ignoring death as an organizing principle. The presented model only incorporates the simplest possible ecological interference, namely limitation of disease spread when host is super-infected by a new diseases. The model will simply assume that a host obtains immunity against any current infection after some time. We do not distinguish between whether the host is actually sick or is just being a passive carrier – both will lead to immunization. The interaction between diseases is subsequently included by decreasing infection probability with number of diseases.

For any host species in the real world there are multiple diseases that compete for it. In this paper we would like to ask how such coupling between diseases influences their diversity through time and space? How does the system size influence the outcome? How would the system behave if the frequency of diseases is much higher than what we observe in our macroscopic world? These questions we will address through a model describing how host immunization against old diseases effectively allocates resources for new diseases.

### Model

A standard extension of more traditional infection-recovery-immunization models [10], [22], [23] would be to include spreading of multiple diseases where each can transmit acquired diseases within a fixed time window 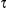 after it became infected. In such models, as well as in real diseases, the length of the infectious period 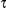 is directly related to the probability that an infected host can spread the disease. If the probability to spread an infection was independent from the number of diseases that the host currently has, then the spread of one disease would be entirely independent of other diseases. That would clearly be unrealistic. However this deficiency could be remedied by letting the infection probability decrease with the number of infections the host has.

In this paper we consider an alternative and simpler way to include interactions between competing pathogens. In our scenario a host can only transmit the last disease it was infected with. In this way we have eliminated the parameter 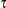 while still incorporating the effect that the more diseases there are in a region, the smaller is the transmission rate of each of them.

Our model considers individual hosts which have only one disease at a time and which each hold a particular disease during *maximally one* continuous time period. This results in “spreading of immunity” as a main dynamical trait of our multi-epidemics model. When infected by a new disease, the host becomes immune to previous diseases and thus *never returns* to any of the infections that he had at earlier times. This is the key element in our simplified model, which, as we will see, predicts a complex landscape of interfering infection waves. The model is governed by only one parameter, the small rate 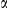 at which new diseases originate in the individual hosts. As the number of possible diseases is in principle infinite, we assume that each new disease appears spontaneously only once.

In this paper we consider epidemics in terms of a minimal model for emergence and spreading of multiple diseases on a 2-d square lattice with 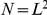 sites each representing a host. We use periodic boundary conditions. Each site 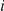 can be assigned a number 
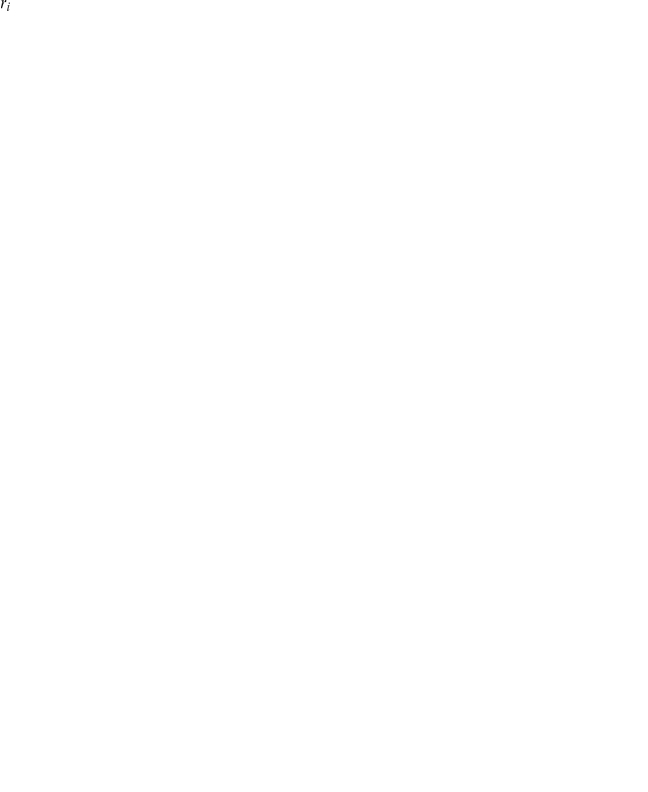
 which can take any integer value. This number plays the role of the present disease. At any time-step one attempts the following two moves:

- Select a random site 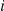 and one of its four nearest neighbors 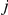. The integer value 
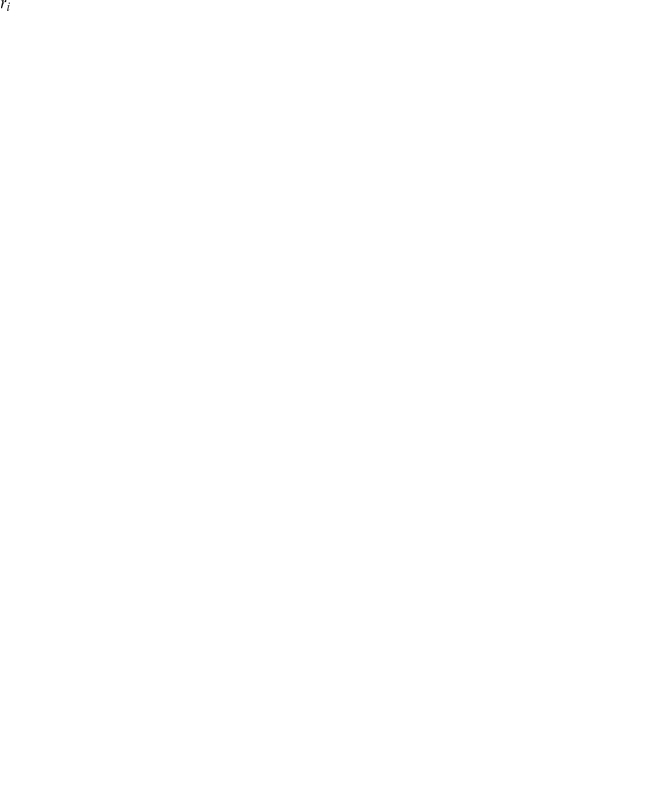
 of site 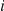 is changed to the value 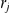 of site 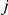, provided that site 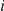 never assumed that particular integer value 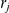 before. In case it had, then no update is made.
- With probability 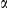 another random site 
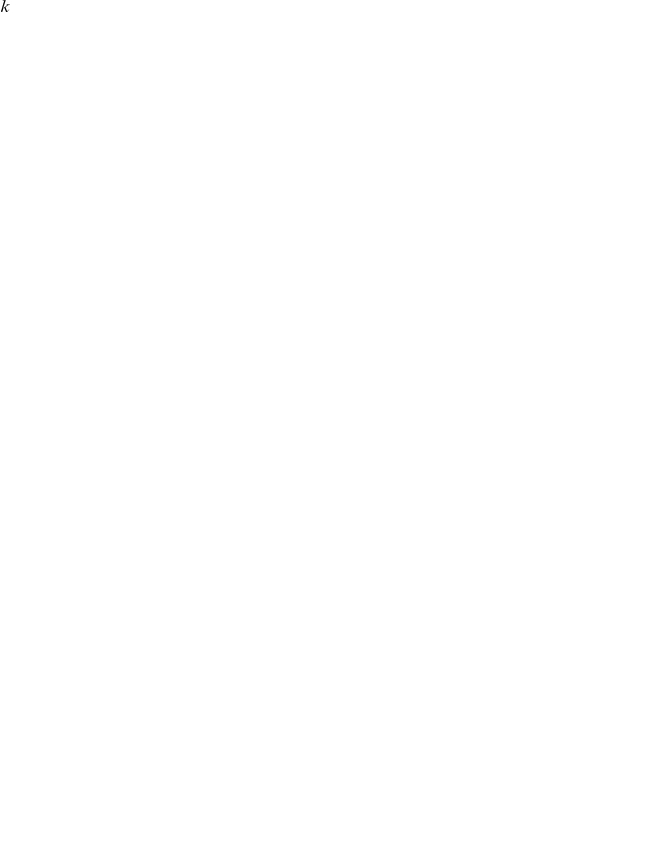
 is selected and assigned a new random integer which does not appear anywhere else in the system. Thus 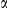 represents the mutation rate for new diseases.

The model can be run online as a java.applet at http://cmol.nbi.dk/models/immunity/Template.html. Notice that the model includes interactions between pathogens, through the assumption that only the last infection of a given host is infectious. When infections happens fast after each other they therefore inactivate each other successively.

A key difference to previous models of disease spreading is the ongoing emergence of new disease and that the presence of several diseases allows one disease to inhibit the spreading of another. This is because 1) any individual host can only have one disease at a time, and 2) hosts are instantly cured from the previous disease by superinfection by another disease. Another key ingredient is the “never return” assumption which means that “older” diseases are always driven into new territories, while being eliminated from old territories. This corresponds to an ongoing spatial “Red Queen effect” [24] where every disease has to keep moving just to maintain its own existence. When a given disease has explored all available space it dies out, very much in accordance with the fate of typical epidemics from human history.

## Results

**Figure 1:**
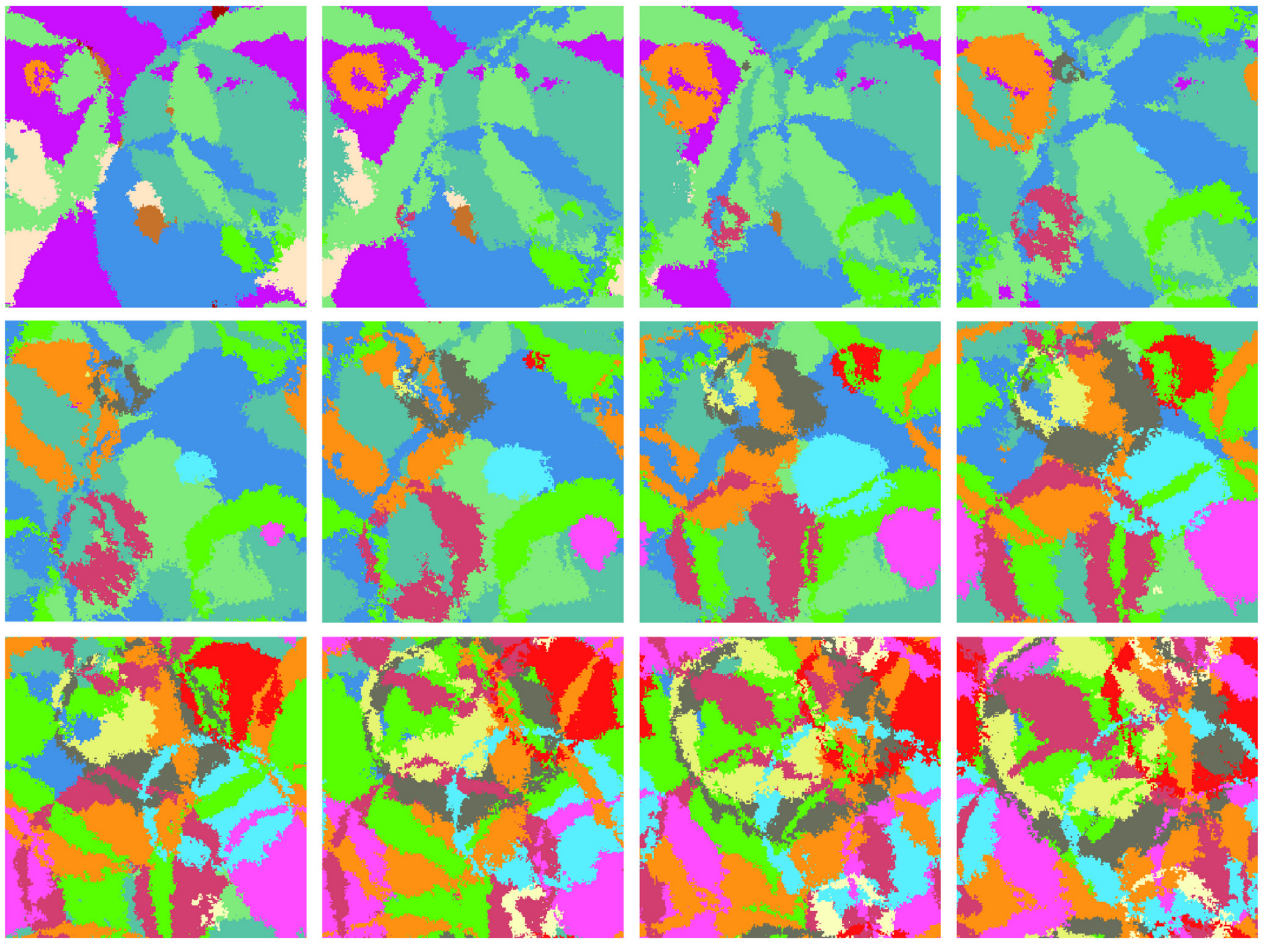
Dynamics of multiple epidemics. 12 consecutive snapshots of a 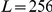 system with 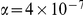. There are 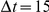 updates per site between the snapshots. Note the wavefronts penetrating each other while the areas left behind the wavefronts are re-colonized from nucleation centers at the colliding fronts.

In Figure 1 we show a typical sequence of snapshots of the model at some intermediate 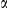 value. One sees a number of “solitary like” infection waves, each of which tends to leave a circular patch with a color that characterizes one particular state. However, other states (diseases) may percolate across any particular infection wave, and re-establish a new state in the otherwise homogeneous patch. This is for example seen by following the growth of the cyan patch in frames 4–7, which is subsequently invaded by the green and orange fronts in frame 7. As a result the propagating “cyan” state is a thin wave whose interior is replaced by “green”, “orange”, as well as other “diseases”. Our model indeed predicts an ongoing battle for survival that requires any particular disease to be constantly “running” in order to maintain existence. Nevertheless, existence of any disease is only temporary, until any available site has become immunized against that particular disease.

**Figure 2:**
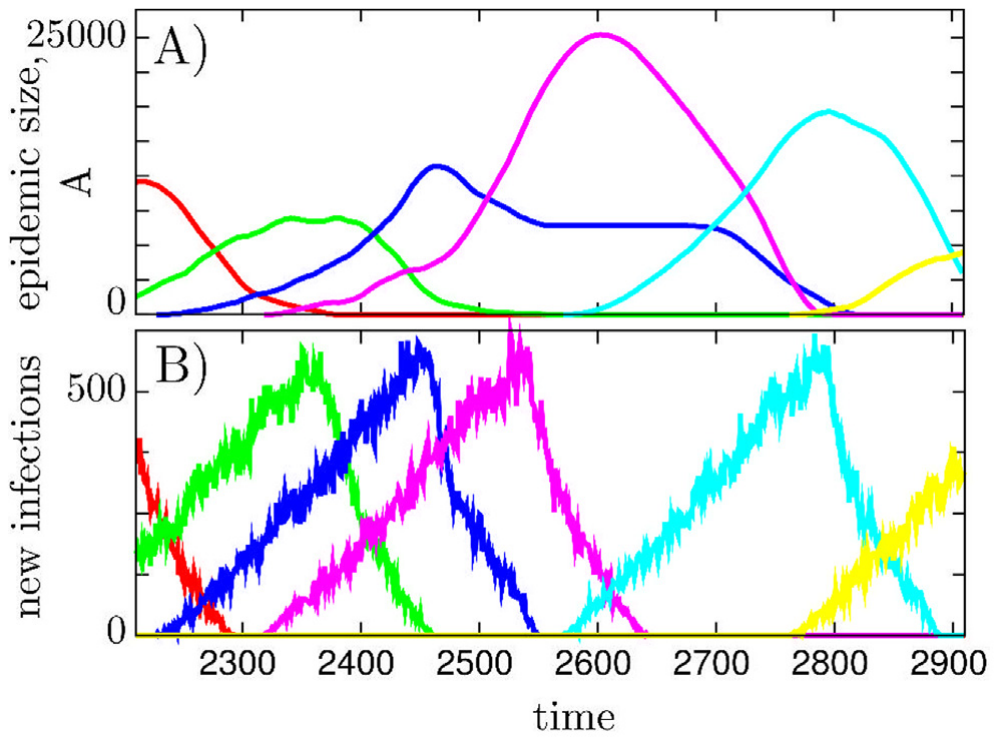
Rise and fall of epidemics. A) Dynamics of a few epidemics quantified through their spatial extent (A). System size and model parameter are the same as in Fig. 3. B) The corresponding rates of infection (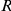) are counted as number of new infections per time-step. The colors in the upper and the lower panels correspond to each other. Note that the total number of infected sites of a given disease can drop while new infections still take place. For larger 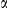, 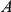 for a given disease goes down whereas its infection rate 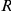 remains of order 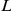 with a time dependence that more closely increases or decreases together with 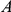.

**Figure 3:**
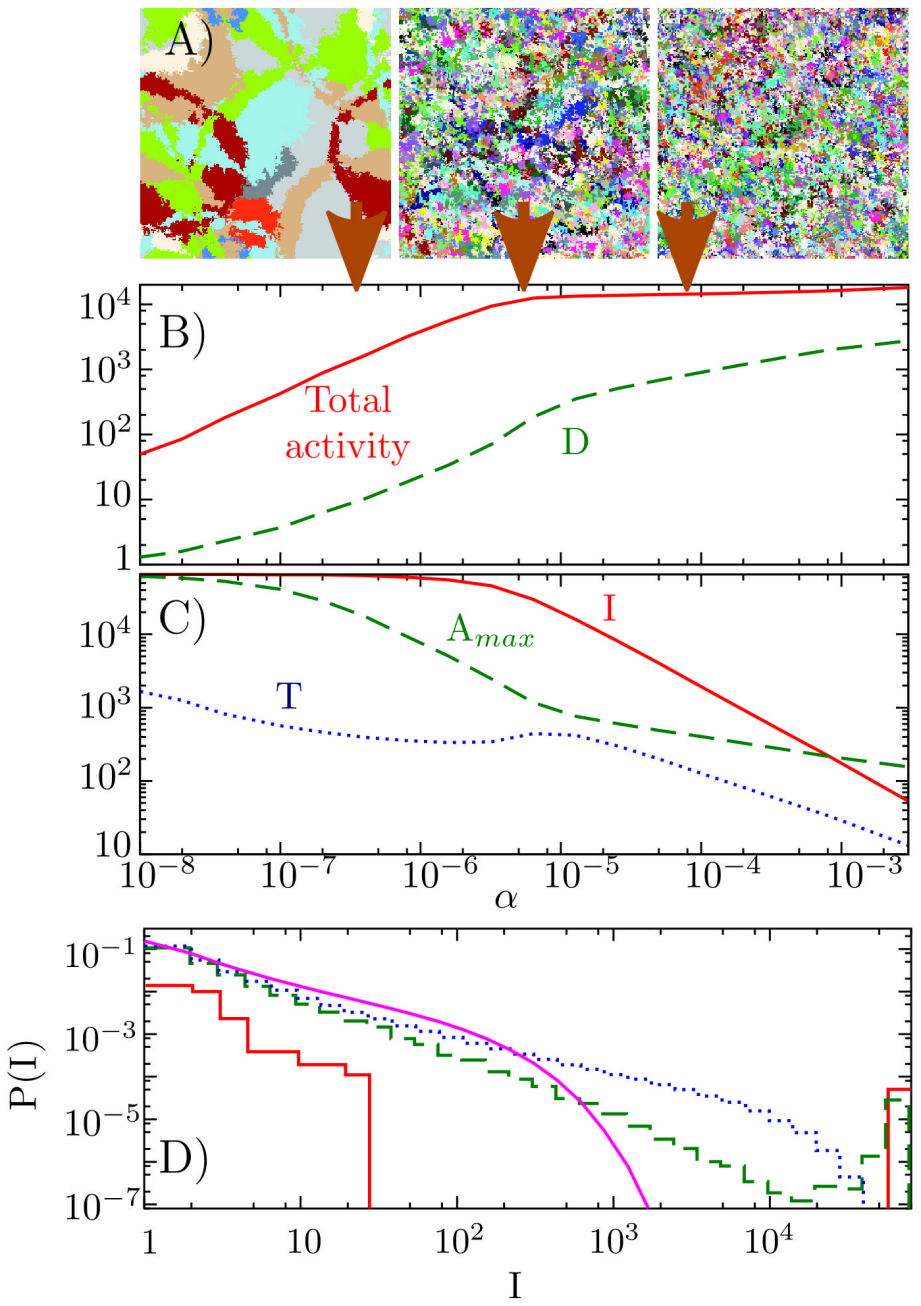
Steady state behavior as function of 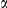 for a 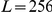 system. A) 3 snapshots, taken at the 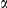 values indicated by the brown arrows. B) Diversity (D) and the total activity, measured as the total number of new infections per time-step in the system. C) Average accumulated immunity per disease 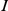, the current disease with maximum extension 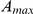, as well as the average duration of diseases (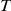). D) Frequency distribution of acquired immunity per disease, for 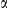 values from top panel, i.e. 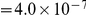 (red), 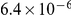 (green dashed), 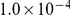 (blue dotted), as well as the very high 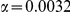 (purple). This corresponds to 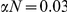, 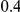, 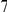, and 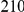 respectively.

The two panels in Figure 2 show epidemic size, A), and activity in terms of number of new infections per time-step B) for some typical diseases in a 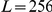 system with 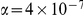. For any particular disease one observes a lifetime of the order of the time it takes to propagate across the system. Also one observes diseases either growing or declining, with a maximum extension that varies substantially between the diseases. However, when counting the total number of infected sites during the existence of a disease, we find that nearly all sites ultimately get infected (see immunity curve, 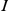 for 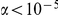 in Figure 3C). By allowing every disease to infect all its 4 neighbors, we are apparently running our model at super critical conditions in spite of the possible eliminations by competitors before replication. For increased 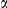, however, many diseases are eliminated relatively fast and the spread of immunity per disease, 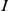, decreases, see Figure 3C.

Figure 2B shows another remarkable “universality” of our model, namely the feature that the frequency of new infections increases linearly in the start of the disease. This feature is closely associated to the linear wave propagation seen in Figure 1, with new infections happening on the rim of the expanding wave. Thus when the wave reaches across the system, the linear increase stops. Obviously, in 3-dimension we would expect a 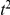 growth, whereas a disease spreading on a random network [25] would grow exponentially with time at its earliest stages. When eventually fitting our model to real data, the average early growth behavior of number of new infections would determine the effective dimension for propagation of the disease.

Figure 3 examines the steady state behavior of the model as a function of the mutation rate 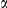. The top panels show representative snapshots of the system, whereas the lower panels show typical characteristics of the system. Notice in particular the linear increase in diversity 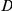 as well as the near constant behavior of disease existence (duration 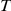) for a wide range of low 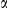 values. At these values there are only few diseases in the system, and any site in the lattice is only rarely infected by a new disease. Thus it appears as if the time interval allocated for spreading of a disease from a particular site is very large. However it is important to understand that in this regime our model effectively works similar to a model with a fixed recovery time of about 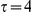 updates. That is, after infection of a site, it takes about 4 updates to attempt to infect all its neighbors on the 2-d square lattice. When infections of all neighbors are attempted, the site can never infect anyone by the current disease and could therefore be viewed as non-infectious.

It is remarkable, that in a large range of 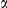 values below 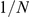, the number of diseases, 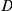, goes up, while the duration, 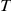 of each of them appears constant. This reflects the fact that although the current spread of each disease obviously has to go down, the infection activity of each of them stays roughly constant. That is, the infection rate approximately constant over a wide range of 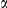 values: The total infection activity (red) with the infection rate (total activity) shown in red in Figure 3B divided by diversity 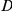 is 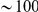, a value that reflects the average linear dimension of an infection front. This is again a reflection of the fact that for these low 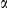 values most sites are neighboring sites with the same disease state and activity only happens at the edges between these homogeneous regions. As 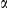 goes up, the infection waves becomes thinner, and start to dissolve the coherently infected regions of the system. This can be followed in the 3 steady state snapshots in Figure 3A.

At intermediate 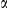, 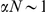, there is of order one disease initiated per update of the whole system. As new diseases typically travel across the entire system, the diversity 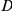 is large, i.e. 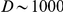, see Figure 3B, and the area allocated to each disease becomes small. On average, only 50–100 sites per disease are observed all of which are active. At this value, most lattice sites becomes exposed to new diseases at every time-step. Accordingly sites become reinfected so fast that they often do not transmit any particular disease: Diseases constantly stop propagation of each other as one disease super-infects the host of the other. The diseases inhibit each others propagation to an extent that limits substantially the spreading of immunization across the system. In Figure 3D we see that for 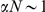, then indeed most diseases only lead to limited immunization (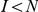), but also that a substantial fraction still succeeds in immunizing across the system 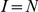.

Finally one may consider 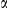 values beyond the limit 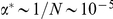. Here the abundance of new diseases seriously influences the spread of old diseases, and immunity against any particular disease diminishes the activity created by the ongoing “turbulence” of new diseases. Remarkably, however, even at quite high 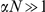, the distribution of immunizations for individual diseases is broad, near scale free, up to a cut-off 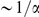 (see Figure 3D). In fact, the probability of a given disease spreading to 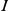 hosts becomes 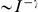 with 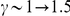, up to a maximal extension given by 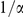. Such wide distribution of accumulated spreading of individuals is close to the 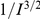 distribution that would be obtained if each propagating disease expanded or contracted as governed by a near critical branching process in infinite dimension [26]. A wide distribution of species or pathogen abundance can alternatively be obtained in multiplicative processes [27].

**Figure 4:**
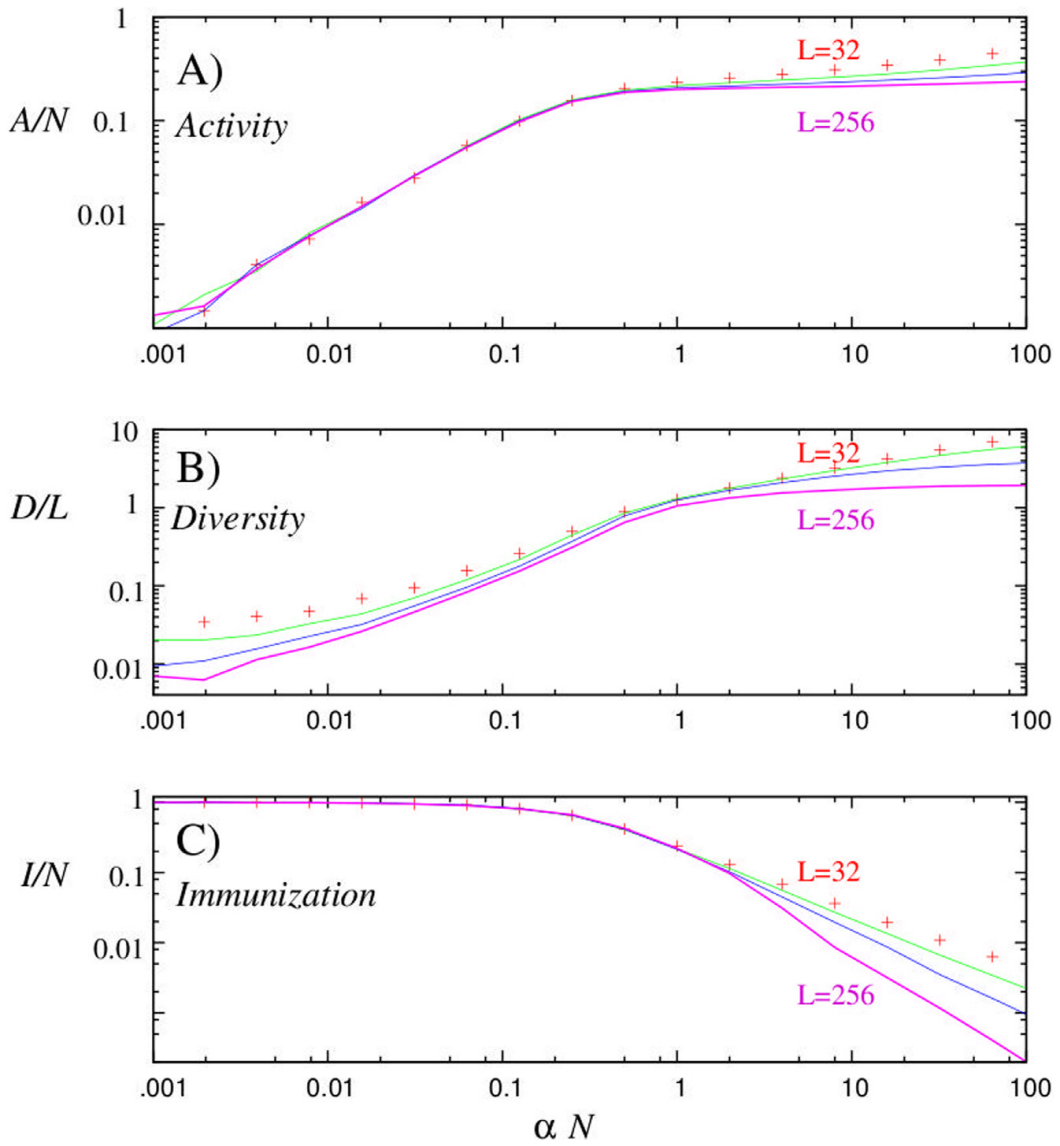
Data collapse of steady state behavior as function of 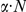, where 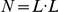 is total number of hosts. Each plot examines the behavior of one particular variable for system sizes 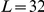 (red crosses), 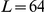, 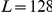, and 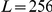 (thick magenta curve), rescaled appropriately. A) Total activity in units of system size, 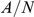, demonstrating a transition to a near saturated regime for 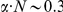. B) Diversity rescaled with linear dimension of system, 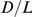. One observes a transition at around 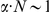 to a near-saturated regime, a saturation that becomes more apparent for large system sizes. C) Average of total accumulated immunity per disease rescaled with system size, 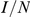. For 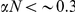 nearly all diseases will spread to all potential hosts. For 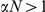 the average spread of diseases decreases with 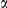.

In Figure 4 we examine systematically how the total activity, the total diversity and the immunization per disease depend on 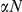, demonstrating a transition between a regime at 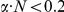 where diseases propagate nearly independently of each other to a regime of strong mutual suppression at 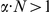.

In the opposite end of the disease activity we consider an extreme limit where no new diseases appear, corresponding to 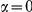. In that case the system will always reach a frozen configuration, a pattern of last non-overruled infections which will depend on the initial distribution of “diseases”. If one starts with a very high number of diseases, the final state is interesting from a complex systems point of view in the sense that the final distribution is scale-free. To investigate this we start from an initial system with maximal diversity of infections across the system.

**Figure 5:**
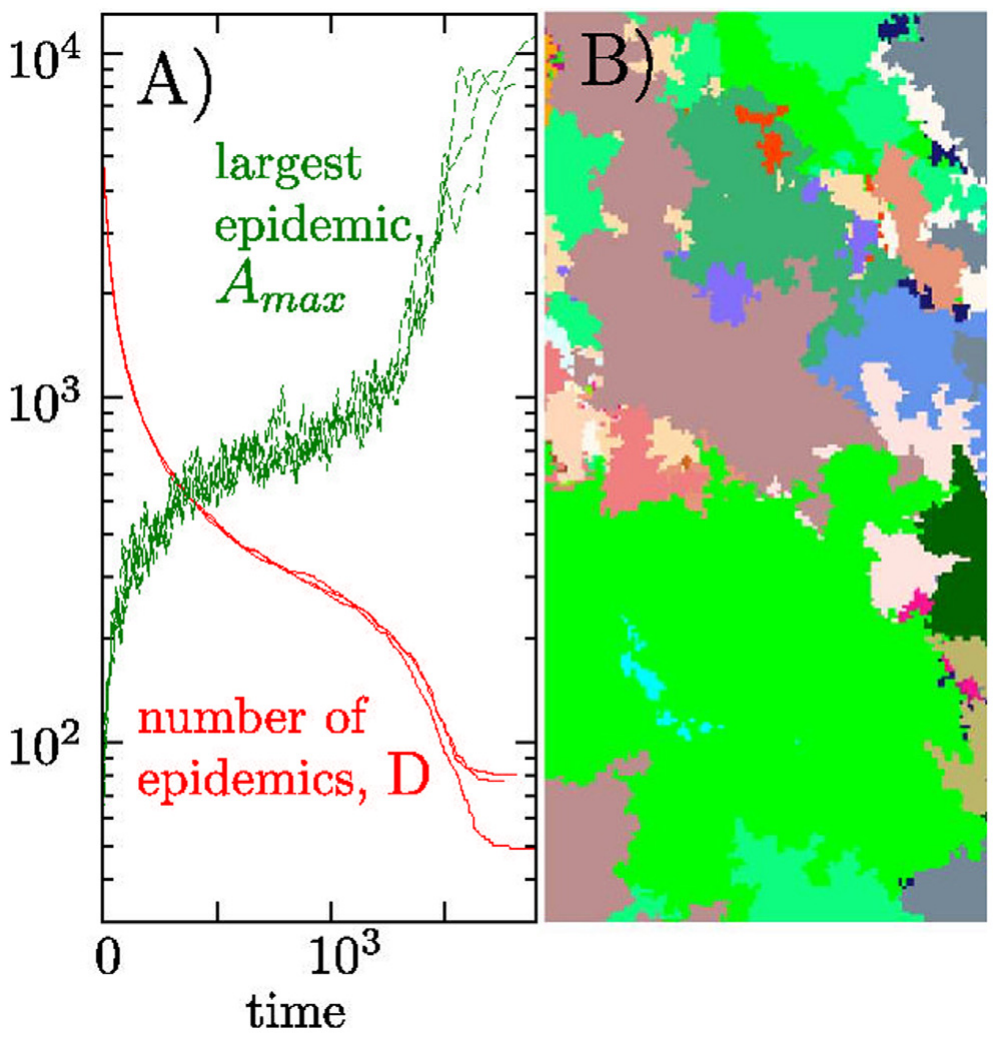
Coarsening dynamics and frozen state. A) Number of remaining diseases 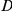 (red color) and the expansion of the dominant disease 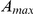 (green color) when relaxing randomized initial conditions at 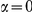 (Initially each site is infected with one of 6000 different diseases). The plot shows 3 independent histories where time 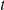 is measured in updates per site. The coarsening in 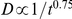 until 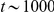, after which the system collapse to its frozen state. The frozen state is reached after a fixation time 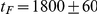 for the shown system size 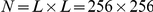. B) Example of one frozen state.

**Figure 6:**
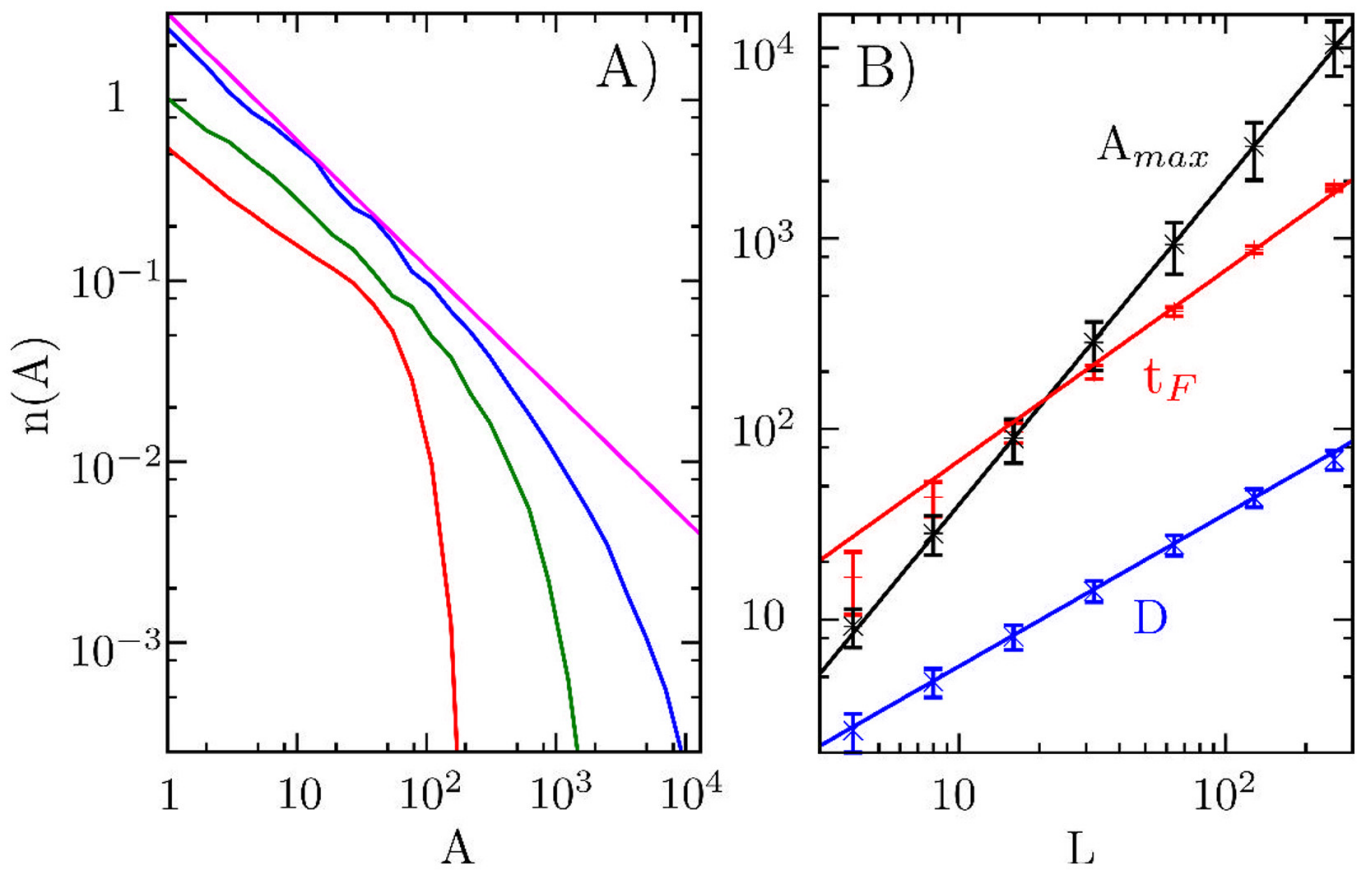
Frozen state scaling. A) Average number of epidemics of various sizes (A) in a frozen state, obtained as in Figure 4. The 3 curves represent system sizes 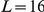 (red), 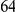 (green), and 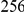 (blue). The straight line shows the scaling 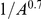. B) Shows the time to reach the frozen state (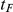), the diversity measured as the number of diseases (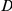), and the size of the largest group (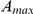) at the frozen state. The lines are fits, 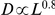, 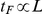, and 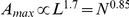, respectively.

That is, all 
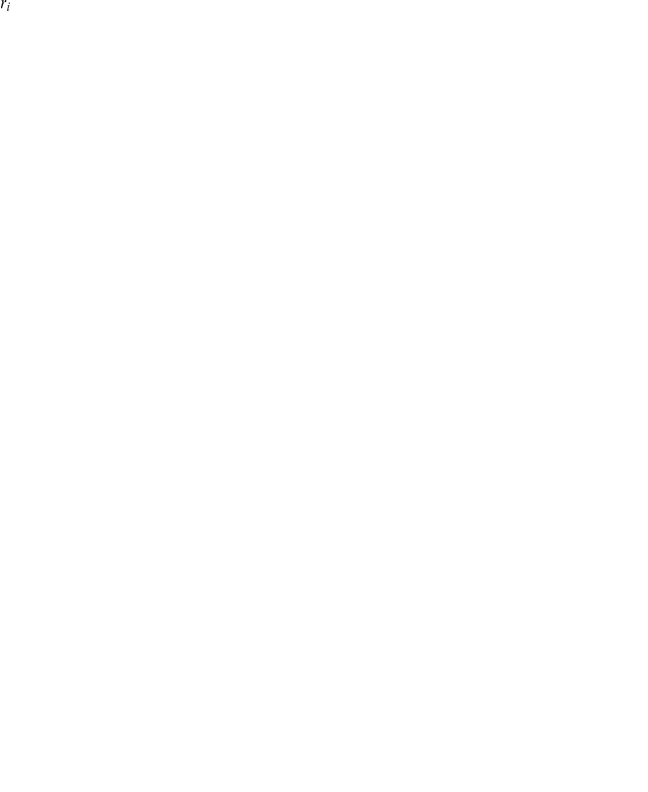
 are assigned different values, representing different infection states. In practice we only assign an initial diversity of 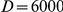, but we have verified that our main results in Figures 5 and 6 do not depend on this number. (coarsening scaling itself, 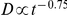, does however require full diversity 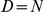 at start).

Figure 6A illustrates the coarsening dynamics towards the frozen configuration. When starting with maximal diversity 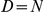, this diversity subsequently decreases as 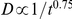. In parallel the dominating state covers an increasingly large area 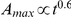 until some collapse time that occurs shortly before the fixation time 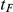. It is remarkable that different realizations reach this frozen state within a narrow time interval around an average that asymptotically approach 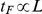: The system freezes when all “diseases” have had sufficient time to propagate linearly across the system.

As seen in Figure 5, the final frozen configuration is characterized by a patchwork of different states. In the framework of our infection model these states represent “diseases” which cannot infect each other, because each of them has already infected and immunized major parts of the system. It is remarkable that the size distribution of these states is exceedingly broad, 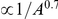, suggesting that the proposed “never return” dynamics opens for a new universality class of coarsening phenomena. A class where the number of different states scales with system size as 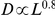, and where the number of sites in the most extended state grows less than the available system, 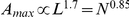, see Figure 6B.

## Discussion

In this paper we propose a minimal description of multiple diseases propagating and interfering through their competition for the host. Its prime benefit is its simplicity in dealing with both immunity and the mutual inhibition between diseases. The model predict two types of spatio-temporal organization: When the number of the diseases is small, we observe a series of inter-penetrating infections, and subsequent immunizations. The epidemic waves resemble patterns of real disease spreading [14]. In contrast, when the number of diseases is large, the waves dissolves into fragmented fronts. The separation of these two regimes is defined solely by the critical value of 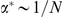.

Our model was primarily inspired by the huge diversity of bacteriophages found in bacteria-phage ecosystems [1], [28]–[31], as well as by a rapid turnover of successive phage infections [4], [32]. In the bacteria-phage interpretation each lattice site in our model correspond to a clonal bacterial colony and the infectious diseases to particular bacteriophages. When a particular phage reaches a colony it kills all but a few mutant bacteria in the colony [33], or, if the phage is temperate [34], it leaves prophages in the host cells. Subsequently these mutants or lysogens grow to reestablish a new colony that will be immune to also this particular phage. As the system becomes exposed to more phages, our model implies that the surviving bacteria obtain more refined defense mechanism. Such an ongoing refinement in practice will be limited by “back mutations” (revertants). Evidence for the long and ongoing battle between phages and bacteria is found in the many elaborate defense mechanism of bacteria against phages (see eg. [2], [35], [36]).

Whereas most diseases spreading on human scale seems limited by host immune system and thus reflect epidemics at low 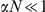, a much lager destructive interference may be found among phages which in soil are reported to differ substantially between places separated by only a few centimeter [37]. We therefore argue that phage-bacteria ecosystems may be characterized by extremely high 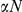, 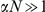. In fact it is tempting to speculate that bacteriophages effectively limit spread of each other by their immense diversity, associated to a very high 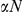.

Overall, we suggest that pathogens spreading on animal or plant hosts behave much like the infection waves seen for 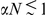, whereas phages preying on procaryotes resemble the multiple fragmented infections that are expected at 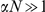.

Our model is indeed hugely simplistic, and naturally invites for consideration of the many other ways that multiple diseases may interact with each other and their hosts immune system. In particular one may consider an immune system of hosts that is limited, one may consider death of the host, one may include diseases that facilitate infections of other diseases, or one may even extend the model to include relationships between subsequent diseases and associated cross immunization.

One particular extension is associated to the tendency of back-mutations, or revertants, for the immunized bacteria in the phage-bacteria ecology. The associated loss of immunization could be included by limiting a given site's immunity to its latest 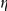 diseases. In this extended model the pattern of infection depends very much on the size of 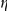 relative to the expected number of diseases in the system for the given 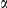. For 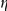 large, the limited immunization is not challenged and the system behaves as in the present model. For lower 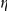 old diseases would be able to re-infect their old hosts and the resulting infection dynamics can become much more chaotic, reflecting an ongoing accumulation of diseases as each of them is able to survive longer by reinfecting old hosts. As a consequence then low immunization (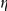) can result in higher disease diversity.

Another feature to be considered is death of the host. The model easily allows extensions where one allows for both, random death of hosts with some background rate, and/or death induced by spreading pathogens. In both cases, new individuals need to be born, in order to sustain the long time survival of the system. If death is unrelated to diseases, and newborns are born without immunity the model correspond to our standard model supplemented with a time limited immunity of the hosts. If, on the other hand, death is associated to diseases, the spatial pattern of disease spreading could be hugely influenced by the self-organized barriers of empty lattice sites caused by death. This in itself calls for a more elaborate numerical study, including a parameter for death rate, and another parameter for the rate at which hosts are reborn in empty sites.

To summarize the weaknesses and strengths of our model, then the strength is its simplicity, the weakness is the multiple real world features of various individual diseases that our model so bluntly disregards. Features like death, limited immunity, cross-immunization [17]–[21], or oppositely of infections that increase the likelihood of subsequent infections (like measles increase likelihood for streptococcal superinfections, or P4-phage that prey on *E.coli* infected by P2-phage [38]). Many of these features can however be considered within our schematic immunization model. A main challenge is to include such effects in a way that is sufficiently robust to the numerous assumptions and parameters of such more elaborate interactions.

## Methods

The model is simulated in both fortran and java programming language, using standard Laptops. It can be run online as a java.applet at http://cmol.nbi.dk/models/immunity/Template.html.
